# Neuroplasticity induced by general anaesthesia: study protocol for a randomised cross-over clinical trial exploring the effects of sevoflurane and propofol on the brain – A 3-T magnetic resonance imaging study of healthy volunteers

**DOI:** 10.1186/s13063-020-04468-y

**Published:** 2020-09-22

**Authors:** Signe Sloth Madsen, Kirsten Møller, Karsten Skovgaard Olsen, Mark Bitsch Vestergaard, Ulrich Lindberg, Henrik Bo Wiberg Larsson, Johan Mårtensson, Mads U. Werner, Sofia Alexandra Gaspar Santos, Mohammad Sohail Asghar

**Affiliations:** 1 Department of Neuroanaesthesiology, The Neuroscience Centre, Rigshospitalet, University of Copenhagen, Blegdamsvej 9, 2100 Copenhagen, Denmark; 2 Department of Clinical Medicine, Faculty of Health and Medical Sciences, University of Copenhagen, Copenhagen, Denmark; 3 Department of Neuroanaesthesiology, Rigshospitalet Glostrup, University of Copenhagen, Valdemar Hansens Vej 15, 2600 Glostrup, Denmark; 4 Functional Imaging Unit, Department of Clinical Physiology, Nuclear Medicine and PET, Rigshospitalet, University of Copenhagen, Valdemar Hansens Vej 1-23, entrance 5, 2600 Glostrup, Denmark; 5 Faculty of Medicine, Department of Clinical Sciences Lund, Logopedics, Phoniatrics and Audiology, Lund University, 22100 Lund, Sweden; 6 Multidisciplinary Pain Center, The Neuroscience Center, Rigshospitalet, Blegdamsvej 9, 2100 Copenhagen, Denmark

**Keywords:** Neuroplasticity, Neuroplastic changes, General anaesthesia, Magnetic resonance imaging, Sevoflurane, Propofol, Healthy volunteers, Consciousness, Cognitive, Fatigue

## Abstract

**Background:**

Although used extensively worldwide, the effects of general anaesthesia on the human brain remain largely elusive. Moreover, general anaesthesia may contribute to serious conditions or adverse events such as postoperative cognitive dysfunction and delirium. To understand the basic mechanisms of general anaesthesia, this project aims to study and compare possible de novo neuroplastic changes induced by two commonly used types of general anaesthesia, i.e. inhalation anaesthesia by sevoflurane and intravenously administered anaesthesia by propofol. In addition, we wish to to explore possible associations between neuroplastic changes, neuropsychological adverse effects and subjective changes in fatigue and well-being.

**Methods:**

This is a randomised, participant- and assessor-blinded, cross-over clinical trial. Thirty healthy volunteers (male:female ratio 1:1) will be randomised to general anaesthesia by either sevoflurane or propofol. Multimodal magnetic resonance imaging (MRI) of the brain will be performed before and after general anaesthesia and repeated after 1 and 8 days. Each magnetic resonance imaging session will be accompanied by cognitive testing and questionnaires on fatigue and well-being. After a wash-out period of 4 weeks, the volunteers will receive the other type of anaesthetic (sevoflurane or propofol), followed by the same series of tests. Primary outcomes: changes in T1-weighted 3D anatomy and diffusion tensor imaging. Secondary outcomes: changes in resting-state functional magnetic resonance imaging, fatigue, well-being, cognitive function, correlations between magnetic resonance imaging findings and the clinical outcomes (questionnaires and cognitive function). Exploratory outcomes: changes in cerebral perfusion and oxygen metabolism, lactate, and response to visual stimuli.

**Discussion:**

To the best of our knowledge, this is the most extensive and advanced series of studies with head-to-head comparison of two widely used methods for general anaesthesia. Recruitment was initiated in September 2019.

**Trial registration:**

Approved by the Research Ethics Committee in the Capital Region of Denmark, ref. H-18028925 (6 September 2018). EudraCT and Danish Medicines Agency: 2018-001252-35 (23 March 2018). www.clinicaltrials.gov, ID: NCT04125121. Retrospectively registered on 10 October 2019.

## Background

General anaesthesia (GA) was introduced successfully more than 150 years ago, and has been a prerequisite for the advancement of modern surgery [1]. Globally, approximately a quarter of a billion major surgeries, and a possibly higher number of less invasive procedures and examinations, require GA every year. In Denmark alone, approximately 200,000 patients annually undergo GA [2]. Although used extensively on a daily basis all over the world, the effects of GA on the human brain remain largely elusive.

### General anaesthesia

Presently, the two main types of GA are: (1) inhalation anaesthesia by a volatile agent, and (2) total intravenous anaesthesia (TIVA). For inhalation anaesthesia, typically a halogenated anaesthetic gas, such as sevoflurane, is administered. To alleviate pain during surgical procedures, an intravenously administered (IV) analgesic is commonly administered (e.g. fast-acting opioids such as remifentanil). For TIVA, both the hypnotic agent and any supplemental analgesics are administered intravenously. Propofol is the most widely used fast-acting hypnotic agent for GA by TIVA. Early studies on the action of anaesthetics have focussed mainly on their molecular and cellular targets. There is consensus that inhaled anaesthetics induce anaesthesia by enhancing inhibitory and attenuating excitatory ion channels in cell membranes. However, the precise mechanism of action, and whether this occurs through direct binding or by physical changes of membranes, is not known [3]. By contrast, propofol appears to activate the inhibitory pathway of the neurotransmitter gamma-aminobutyric acid (GABA) through receptors located predominantly in the parietal cortex and thalamus [4–6]. The hypnotic effect may be caused by interaction with other neurotransmitters [6]. The effect of GA is dose-dependent. Initially, working memory is suppressed. As the dose is increased, consciousness and voluntary responsiveness begin to fade, while some autonomic processes remain operational until very high doses are reached [7–9]. It is well-known that GA does not induce unconsciousness simply by a widespread, non-specific suppression of neural activity [10]. However, the specific neural mechanisms underlying amnesia and loss of consciousness, including when these phenomena are induced by GA, remain controversial [11–13]. In addition, which method for GA (inhaled anaesthetics vs. TIVA) that should be preferred is a topic of ongoing discussion among anaesthesiologists [14].

### Surgical stress and cognitive effects

The surgical trauma together with GA can elicit profound changes in the endocrine, immune and nervous systems, collectively known as the surgical stress response. The pathophysiology of this stress response is characterised by activation of the hypothalamic-pituitary-adrenal-axis and increased sympatho-adrenomedullary activity. This leads to increased secretion of stress hormones, such as cortisol and pro-inflammatory cytokines, as well as altered autonomic nervous system activity and immune function. While ideal for overcoming physiological stress, hyperactivation may ultimately cause dysfunction of one or more organs including the central nervous system (CNS). The response may manifest as increased pain, disturbances in the circadian rhythms, as well as impairment of memory and executive functions. Post-operative cognitive dysfunction (POCD) is associated with increased mortality and increased risk of leaving employment prematurely [15]. Post-operative delirium (POD) is characterised by fluctuating levels of attention and consciousness. Associated with both longer hospitalisation, poor outcome, and increased early mortality, POD is a serious condition [16, 17]. General anaesthesia alone may also trigger a stress response in the CNS [18]. Both POCD and POD are well-described among older-age patients [19]. A Cochrane review of the clinical studies focussing on older-age non-cardiac surgery patients receiving inhalational vs. IV GA did not find significant difference in the post-operative cognitive status of the two groups [20]. However, very few studies have explored the effects on cognitive performance resulting from GA in younger adults [21–23]. To the best of our knowledge, the isolated effects from GA with inhalation anaesthesia vs. TIVA have never been investigated.

### MRI

Brain imaging has contributed substantially to our current understanding of brain structure and function. Structural magnetic resonance imaging (sMRI) provides information about microstructural changes in brain anatomy [24], while functional MRI (fMRI) permits measurements of neuronal activity during stimulation [25, 26] and at rest (= resting-state fMRI (rsfMRI)) [27]. Moreover, MRI may be used to investigate cerebral haemodynamics and for pharmacological imaging (the interaction between brain physiology, neuronal activity and pharmacological agents). Thus, MRI offers ideal opportunities to study the in vivo effects of GA on the human brain. However, only a very limited number of studies have been published within this field. No studies have examined possible neuroplastic effects of GA on the brain by advanced neuroimaging techniques such as MRI or positron emission tomography (PET). Most of the studies focus on changes in regional cerebral blood flow or neuronal activity during sedation with propofol [28–31]. Mhuircheartaigh et al. found decreasing activity with increasing depth of propofol sedation in areas corresponding to the stimulus applied during propofol sedation: the anterior insula, anterior cingulate cortex and superior temporal gyrus during auditory stimulation, and in the putamen and globus pallidus (contralateral), and insula (bilateral) in response to noxious stimulation on the left hand. Furthermore, they found that propofol sedation impairs the function of basal ganglia circuits and thalamo-cortical connectivity [30]. The same research group described similar findings in another study, including impaired thalamo-cortical networks [31]. Two MRI studies focussed on memory functions during propofol sedation: Quan et al. found inhibited activation of the superior and middle temporal gyri, and inferior parietal lobuli with auditory stimuli [29], and Pryor et al. found suppression of the hippocampal response to visual stimuli [28]. Four studies in which PET scans were performed during propofol sedation suggested impaired thalamocortical connectivity and decreased activity in the cerebellum [32–35]. In addition to these findings, Schlunzen et al. found metabolic and vascular depression in the cortex and thalamus [36]. Thus, propofol sedation in subanaesthetic doses seems to impair the thalamocortical network. Three studies have used fMRI to assess changes in regional cerebral blood flow (CBF) in response to mild sevoflurane sedation [37–39]. One study evaluated CBF during visual, auditory and motor activation, and found a significant decrease in CBF in the primary and secondary visual cortices, thalamus, hippocampus and supplementary motor area with sevoflurane [37]. A comparable study showed similar findings regarding the visual and auditory cortical regions [38]. However, the third study found that sevoflurane increased both regional cerebral blood volume and CBF in the striatum, thalamus and frontal, parietal and occipital cortices [39]. Only a single study utilising resting-state fMRI (rsfMRI) has evaluated sevoflurane in doses equalling clinical GA, and found a general reduction in functional connectivity maps (motor cortices) of 78–98% compared to the awake state [40]. Thus, sedation with sevoflurane in subanaesthetic doses may alter the cerebral haemodynamics, whereas GA with sevoflurane (in higher doses) seems to impair cortical connectivity. However, the research in this area remains very limited. As for comparing GA with sevoflurane vs. propofol, a small PET study with eight volunteers found a comparable overall reduction in CBF and oxygen metabolism for both types of anaesthesia, with a slightly less pronounced effect of sevoflurane [41].

Overall, these explorative functional studies were conducted using varying levels of sedation and included very small sample sizes. The stimulation and chosen cerebral regions of interest varied greatly. While they do suggest that pharmachological sedation might be associated with a reduction in the thalamocortical network, they are hardly comparable or collectively conclusive regarding GA in clinically relevant doses. Importantly, possible structural changes in the brain induced by GA remain unexplored.

In this present study, we want to focus on the effects of the two main anaesthetic agents used for GA in healthy volunteers, without the interactions and confounders of polypharmacy frequently found in patients scheduled for GA on clinical indications. Multimodal imaging by MRI will be used to study the structural and functional effects of the anaesthetic agents on the brain. In addition, neuropsychological testing will be performed to assess the impact on cognitive performance resulting from the anaesthetic agents. Furthermore, we will use questionnaires to measure changes in self-reported fatigue and general well-being following GA. Thus, the effects induced by isolated exposure to GA by sevoflurane vs. propofol can be explored and described.

### Aim

1. To explore and compare possible de novo neuroplastic changes induced by GA with a volatile agent (sevoflurane) and TIVA (propofol), respectively
2. To elucidate possible associations between MRI findings and clinical outcomes

### Hypotheses

The hypotheses of this study are that:

- Compared with baseline, GA by propofol or sevoflurane is associated with (a) focal micro-structural neuroplastic changes (volume and morphology), (b) changes in white-matter tracts and (c) changes in resting- state neuronal connectivity, as detected by MRI
- General anaesthesia by sevoflurane is associated with more pronounced changes in neuroplasticity compared with GA by propofol
- Compared with baseline, GA using propofol or sevoflurane is associated with reduced self-rated well-being (increased fatigue and reduced general comfort) and executive cognitive functions; a more pronounced reduction is observed after sevoflurane compared with propofol
- The presence and severity of structural MRI changes after GA are correlated to changes in cognitive performance as well as self-rated measures of fatigue and well-being

## Methods

### Study design

This study is a participant- and assessor-blinded, randomised, cross-over clinical trial. The study population is healthy volunteers with a male:female ratio 1:1. Inclusion will continue until 30 volunteers have completed the study (See Table 1 for inclusion and exclusion criteria). The intervention is 2 h of GA. On each of two intervention days, the volunteer will be exposed to GA by either TIVA (propofol) or by a volatile agent (sevoflurane). Each volunteer will receive both types of GA during the study, the order being determined according to randomisation. Participants will be blinded to the type of GA. The assessment of outcome will be blinded during analysis.

**Table 1:** Inclusion and exclusion criteria

**Inclusion criteria**	**Exclusion criteria**
• Age ≥ 18 and ≤ 35 years • Healthy individual • BMI ≥ 18 kg/m^2^ and ≤ 30 kg/m^2^ • Normal electrocardiogram (ECG) • Normal physical examination, including neurological examination, auscultation of the heart and lungs, and measurement of blood pressure and pulse • American Society of Anaesthesiologists (ASA) class 1 [44] • Mallampati I-II and simplified airway risk index (SARI) 0–2 (i.e. no indication of difficult intubation). See Tables 2 and 3 for details • Right-handed • Female participants must use safe contraceptives (hormonal or mechanical, including IUDs) • Speaks and understands Danish • Provides oral and written informed consent	• Contraindications to MRI (as described below) • Left-handedness or ambidexterity. • History of complications to general anaesthesia, including malignant hyperthermia • Family history of malignant hyperthermia • Known incident of malignant hyperthermia or any unexplained complication to general anaesthesia among close relatives • Allergy to any kind of medication or material to which the volunteer could be exposed during this study • History of serious illness • History of cancer, immune disease, autoimmune disease, chronic pain or psychiatric illness • History of neurological disease with permanent neurological deficits or ongoing neurodegeneration • Major trauma or head trauma with any symptoms present at the time of inclusion • Surgery less than 6 weeks prior to the study period • Infection (with fever) less than 2 weeks prior to or during the study sessions • Daily use of any medication (not counting contraceptives) • Consumed anti-depressants during the last 30 days before study days • Weekly intake of > 21 (for women > 14) units of alcohol • Heavy intake of caffeine (> 5 cups/day) • Smoking during the last 30 days before study days • Substance misuse (assessed by the investigator) • Pregnancy • Reflux or dyspepsia • Poor dental status or oral health • Expected or suspected difficult airway • Declines receiving information regarding accidental pathological findings during MRI scans of the brain • Cannot cooperate to tests • Otherwise judged unfit for participation by the investigator
	Exclusion criteria during the study (leading to withdrawal)
	• Any of the above-mentioned exclusion criteria • Major trauma or head trauma during the study period • Surgery during the study period • Infection (with fever) during the study period • Consumption of more than 3 units of alcohol within 24 h before each study day (intervention day or MRI scan day) • Consumed analgesics within 3 days before each study day • Consumed anti-histamines less than 48 h before each study day • Intake of caffeine 12 h prior to each study day • Smoking

*BMI* body mass index, *IUD* intrauterine contraceptive device, *MRI* magnetic resonance imaging

**Contraindications to MRI** Contraindications to MRI include the following: severe claustrophobia rendering the subject unable to undergo MRI without administration of one or more sedatives or anaesthetics, pacemaker implant, artificial heart valve, cochlear/stapes prosthetics, irremovable insulin pump, neurostimulator, metal clips from previous surgical procedures, other metallic foreign objects, shrapnel or shell splinter, the presence of intravascular catheters other than a peripheral intravenous catheter, shunts and drainage tubes

**Table 2:** Mallampati classification

**Class**	**Visible parts by inspection of open mouth**
I	Soft palate, fauces, tonsillar pillars and uvula
II	Soft palate, fauces and uvula
III	Soft palate and base of uvula
IV	Soft palate invisible

**Table 3:** Simplified Airway Risk Index (SARI)

**Parameter**	**0 Points**	**1 Point**	**2 Points**
1. Mouth opening	> 4 cm	< 4 cm	–
2. Thyromental distance	> 6.5 cm	6–6.5 cm	< 6.5 cm
3. Mallampati class	I or II	III	IV
4. Neck movement	> 90°	80–90°	< 80°
5. Underbite	Can protrude jaw	Cannot protrude jaw	–
6. Body weight	< 90 kg	90–110 kg	> 110 kg
7. Previous intubation history	No difficulty	Unsure or unknown	Known difficulty
**Total score ≥ 4 predicts difficult intubation**			

### Outcomes

Outcome parameters will be measured before and after GA, and subsequently after 1 and 8 days. Possible differences can, thus, be assessed before and after GA, within each type of general anaesthesia, and between GA methods (sevoflurane vs. propofol).

#### Primary outcome

1. Volume and morphology of the brain’s anatomical structures as recorded by T1-weighted three-dimensional (T1w3D) anatomical MRI scans, and white-matter microstructure as measured using diffusion tensor imaging (DTI)

#### Secondary outcomes

1. Differences in resting-state functional MRI (rsfMRI)
2. Severity and characteristics of fatigue, as measured by Multidimensional Fatigue Inventory (MFI-20) [42]
3. Cognitive function including attention, speed, and executive functions as measured by computer-based neuropsychological tests (Paced Auditory Serial Addition Test (PASAT), Test of Attentional Performance (TAP) and Conners Continuous Performance Test, 3rd edition (CPT3))
4. Quality of Recovery – 15-item questionnaire (QoR) [43]
5. Correlations between MRI findings as well as clinical outcomes as described above

#### Exploratory outcomes

1. Global and regional cerebral perfusion and oxygen metabolism (CMRO_2_) as measured by Phase Contrast Mapping (PCM) and dual-echo arterial spin labelling (ASL)
2. Cerebral metabolism as measured by MR spectroscopy (MRS) of metabolites such as lactate
3. Response to visual stimuli measured by Blood-Oxygen-Level-Dependent (BOLD) signal

### Setting

This study was scheduled to start on 1 September, 2019 and to be completed by 15 December 2020. It will take place at Rigshospitalet Glostrup, a part of Copenhagen University Hospital. Physical examinations, cognitive tests, questionnaires, GA and post-anaesthesia care will take place at the Department of Neuroanaesthesia. The MRI will take place at the Functional Imaging Unit, Department of Clinical Physiology, Nuclear Medicine and PET, Rigshospitalet Glostrup.

### Study setup

#### Inclusion and exclusion criteria (Table 1)

### General procedure

**Fig. 1:**
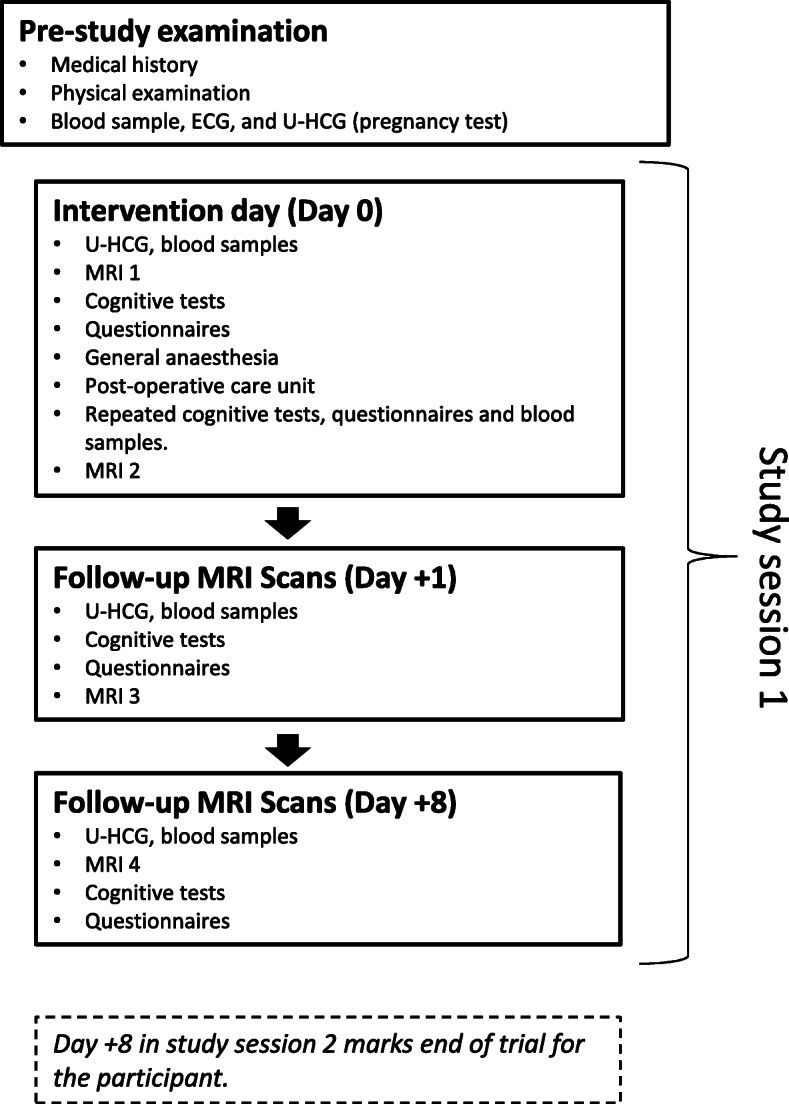
Flow chart for study sessions. Study session 2 mirrors session 1, the only difference being the type of GA on the intervention day (sevoflurane vs. propofol)

**Fig. 2:**
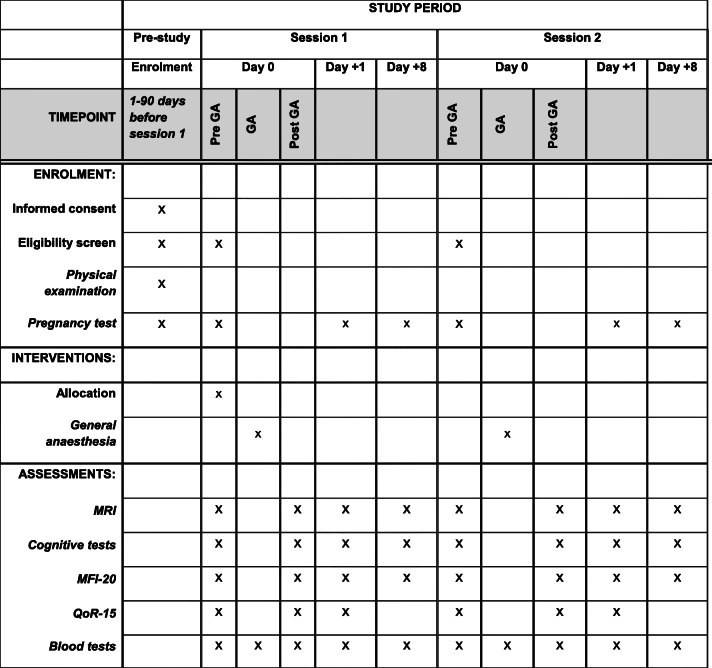
Schematic presentation of the study elements

The study consists of an inclusion interview with a pre-study examination and two separate study sessions separated by 4 weeks. Each study session consists of one intervention day followed by two MRI days. See Fig. 1: Study flow chart for study sessions. Study session 2 mirrors session 1, the only difference being the type of GA on the intervention day (sevoflurane vs. propofol). Figure 2 is a populated Standard Protocol Items: Recommendations for Interventional Trials (SPIRIT) Figure, with a schematic presentation of the study elements.

### Pre-study

#### Recruitment and inclusion

Participants will be recruited in Denmark by online advertisement and via magazines for medical students at the University of Copenhagen. Informed written consent from all volunteers will be obtained by a physician before inclusion in the study. The volunteer will be screened for eligibility according to the inclusion and exclusion criteria. Physical examination, airway assessment (pre-anaesthesia evaluation, including Mallampati- and Simplified Airway Risk Index (SARI)), electrocardiogram (ECG) and blood sampling (haemoglobin, sodium and potassium levels) are carried out. The volunteer will be informed that participation is voluntary and that they may withdraw from the study at any given time. Inclusion and physical examination are done by a physician. Informed consent for any ancillary studies will also be obtained by a physician. See Table 1 for inclusion and exclusion criteria.

#### Randomisation and blinding

This is a participant- and assessor-blinded study, whereas the personel involved in the maintenance of GA are unblinded regarding the anaesthetic agent. The order of sevoflurane or propofol is randomised by a random allocation sequence (simple randomisation), computer-generated by inhouse expertise and stored in sealed and opaque envelopes to secure adequate allocation concealment. Evaluation of data will be done separately, pseudonymised and without information regarding the GA. The questionnaires, MFI-20 and QoR, and the cognitive tests, are presented to the volunteers before randomisation and will be filled out by the volunteer without intervention from anyone having information regarding the GA.

### Study session 1

#### Intervention day (day 0): pre-intervention procedure

The volunteer will be re-screened according to all inclusion and exclusion criteria. It will be ensured that the volunteer is fasting according to the recommendations of The Danish Society for Anaesthesia and Intensive Care Medicine (DASAIM), which are aligned with the guidelines from the European Society of Anaesthesiologists [45]; thus, 2 h of fasting are required for clear liquids and 6 h for all other oral intake. After changing into hospital clothing, baseline MRI scans will be performed (MRI 1). Cognitive testing will be performed, and the questionnaires MFI-20 and QoR will be filled out by the volunteer.

#### General anaesthesia

The principal investigator and the specialist anaesthetic nurse will open the sealed envelope corresponding to the relevant session, and thereby assign the volunteer to GA with either sevoflurane or propofol, depending on randomisation. The volunteer remains blinded to GA type. The volunteer is placed on an operating table in the supine position, carefully adjusted to avoid localised pressure injuries during the GA. Monitoring equipment will be set up for continuous monitoring of heart rate (three-lead ECG) and peripheral oxygen saturation (pulse oximetry). Non-invasive blood pressure is monitored intermittently every 5 min. Body temperature is measured every 30 min with an ear probe. A heating blanket is used if the body temperature drops below 36.0 °C and turned off if the body temperature rises above 37.5 °C. Following intubation, end-tidal CO_2_ (ETCO_2_) is monitored by capnography. Two peripheral intravenous catheters will be inserted.

##### Induction

Regardless of the randomisation, GA is induced by an IV injection of propofol. To facilitate intubation, remifentanil will be infused simultaneously. Propofol and remifentanil will be infused by infusion pumps (B Braun Perfusor Space, B. Braun Melsungen AG, Hessen, Germany) set on target-controlled infusion modus (TCI). Both propofol and remifentanil dosage will be directed by the calculated effect-site concentration (i.e. the concentration of each type of medication in the brain). Propofol infusion rates will be calculated by Schnider’s pharmachokinetic model for propofol [46–48]. This will correspond to a propofol dose well within the routinely used range for induction of 1.5–2.5 mg/kg. Remifentanil infusion rates will be calculated by the Minto model [46]. This will correspond to a remifentanil dose within the routinely used range for intubation of 2.4–4 μg/kg body weight. The infusion rates will thus be based on age, gender, weight and height of each volunteer. TCI with effect-site concentration targets for both propofol and remifentanil ensures the correct infusion rates to obtain and maintain the intended concentrations of the medications at the effect-site (the brain) at any time during the GA. If necessary, additional boli of propofol 20 mg can be injected. Intubation will be performed by video laryngoscopy (McGrath™ MAC, Aircraft Medical Ltd., Edinburgh, UK). After intubation, remifentanil will be discontinued.

##### Maintenance

According to the randomisation, GA will be maintained either via continuous inhalation of sevoflurane or via infusion of propofol. Sevoflurane will be administered via the evaporator system on the ventilator (Dräger Primus®, Drägerwerk AG & Co, Lübeck, Germany). Sevoflurane dosage will be adjusted to 1. 5 mimimum alveolar concentration (MAC) or lower, which equals the routinely used dosage of sevoflurane for maintenance of GA. Infusion of propofol will be administered via the infusion pump (B Braun Perfusor Space, B. Braun Melsungen AG, Hessen, Germany). The infusion pump will continue the TCI directed by effect-site concentration using Schnider’s pharmacokinetic model for propofol, as described under ‘Induction’. This would correspond to a propofol dosage well within the recommended range for maintenance of anaesthesia with propofol (4–12 mg/kg body weight/h). However, higher infusion rates may occur, in particular during induction of GA. Both sevoflurane and propofol doses can be adjusted according to the clinical response of the volunteer. To ensure sufficient depth of GA, bispectral index (BIS) will be monitored and kept within the generally accepted values for GA (40–60) by adjusting the dosage of the anaesthetic agent. In addition, a brief test of response to benign pain will be carried out every 15 min: Pressure applied to the fingernail bed with an algometer (Somedic Algometer type II (Somedic SenseLab AB, Sösdala, Sweden)), and ulnar-nerve stimulation (post-tetanic count) with a standard neuromuscular monitor (Philips NMT on Intellivue, MP70 Anaesthesia). If the volunteer responds with any sign of pain perception (movement, lacrimation, or increase of heart rate or blood pressure), dosage of the anaesthetic agent will be increased, and the test repeated after 5 min. The volunteer will receive a background infusion of NaCl 0.9% during the induction and maintenance phases of GA. The dosage of NaCl will be well within the routinely administered range of 300–500 ml in total during the GA. Ventilation will be maintained via a ventilator (Dräger Primus®, Drägerwerk AG & Co, Lübeck, Germany), on ventilation modus volume autoflow. Inspiratory oxygen will be set between 30 and 45%, respiratory frequency (RF) 10–20/min and tidal volume of 6–10 ml/kg/min with a maximum peak pressure of 30 cmH_2_O. Optimal respiration will be ensured through continuous measurement of end-tidal CO_2_ (ETCO_2_ = 4.0–5.7 kPa), oxygen saturation (SpO_2_ = 94–100%) and airway pressures (PEEP 5–15 mmHg, peak pressure 30 mmHg). GA will be maintained for 2 h after intubation and terminated by stopping the administration of sevoflurane or propofol.

##### Extubation

The volunteer is expected to be ready for extubation at 10–30 min after discontinuing propofol or sevoflurane. They will then be transported to the post-operative care unit.

##### Post-intervention care

In the post-operative care unit, the volunteer will be continuously monitored with heart rate and SpO_2_, while blood pressure will be measured every 15 min. The volunteer will receive oxygen supplementation if needed. Monitoring, care, discharge and intake of food and drinks will adhere to DASAIM’s standard of post-operative care [49, 50]. The volunteers will be observed for at least 2 h. Then, cognitive testing and questionnaires will be repeated before MRI 2. After MRI 2 the volunteer will be allowed to leave the hospital, but will be instructed not to remain alone for the next 24 h as recommended by the American Society of Anaesthesiologists (ASA) [51].

##### Follow-up MRI scan day (+ 1 day)

One day after GA cognitive testing, questionnaires and MRI scans (MRI 3) will be repeated.

##### Follow-up MRI scan day (+ 8 days)

Eight days (7–10) after GA cognitive testing, questionnaires and MRI scans (MRI 4) will be repeated once more.

### Study session 2

Study session 2 will be initiated no earlier than 4 weeks after study session 1 (4 weeks after the intervention day), to ensure a sufficient wash-out period. Before the initiation of study session 2, the volunteer will be re-screened to ensure eligibility for continued participation. Session 2 will be identical to session 1, except that general anaesthetic will be changed from propofol to sevoflurane or vice versa.

#### Procedures for handling adverse effects during the clinical study

##### Monitoring of adverse effects

The volunteers will be monitored for possible adverse effects during GA and in the post-intervention care phase. Any adverse effects will be treated immediately, and the volunteer will be observed until the adverse effect is no longer present. If relevant, the volunteer will be referred to follow-up by a general practitioner. Thus, there will not be a follow-up for the known adverse effects listed below in the setting of this study. The volunteers will be advised to report any medical events during each of the study sessions (from the intervention day until completion of MRI 4 (7–10 days later). In the period between study sessions 1 and 2, the volunteers will not be monitored in any way. However, when registering for study session 2, the volunteers will be asked of any health or medically related events or changes since study session 1, cf. exclusion and withdrawal criteria.

##### Allergies and anaphylaxis

Any volunteer with known medical allergies will be excluded from the study. Should symptoms or suspicion of allergy including anaphylaxis arise, exposure will be stopped immediately, and the condition treated according to already implemented practice in the Department of Neuroanaesthaesiology, Neuroscience Centre, Rigshospitalet. The volunteer will be withdrawn from the study after relevant care, informed of the event and referred to the Danish Anaesthesia Allergy Centre for relevant follow-up. The suspected agent will be registered in the volunteer’s medical journal to avoid future exposure.

##### Hypotension

Hypotension will be defined as a mean arterial pressure (MAP) below 50 mmHg, or a 30% decrease from pre-induction blood pressure. In case of hypotension during GA, the blood pressure will be corrected by the following standard procedures in prioritised order: Trendelenburg position, IV fluid bolus of 300 ml NaCl, IV ephedrine 5–10 mg, IV phenylephrine 0.1 mg. Fluid, ephedrine and phenylephrine can be repeated as in standard procedures already implemented in Department of Neuroanaesthesiology, Neuroscience Centre, Rigshospitalet. In the case of persistent hypotension without effect of these means, anaesthesia will be terminated, and the volunteer withdrawn from the study after relevant care. The volunteer will be informed of the event and referred to a general practitioner for further assessment to ensure diagnosis and treatment of any underlying cause.

##### Pain

During the study mild pain may arise, which can be treated with paracetamol 1000 mg. Severe pain will be registered as an adverse event (AE) and the cause will be analysed to prevent recurrence. The participant will be treated according to established guidelines for managing severe pain in the Department of Neuroanaesthesiology.

##### Nausea and vomiting

Nausea and vomiting may occur as an adverse effect of the anaesthetic agents. In severe cases, this will be treated with ondansetron 1 mg which can be repeated if necessary, to a maximum of 8 mg. In the rare case of treatment failure by ondansetron, dehydrobenzperidol 0.625–1.25 mg can be administered.

### Adherence strategy and discontinuation of the study

The volunteers will be in close contact with the principal investigator throughout the study. Reminders of appointments and information will be sent by e-mail or SMS, and the volunteers will receive telephone calls for extra safety during the study sessions. The volunteers can reach the investigators by telephone and e-mail and are urged to call the investigators immediately at any time if questions, discomfort or any medical symptoms arise. In case of acute emergency, the volunteers should call the public emergency telephone number directly. If a volunteer does not complete the trial, an account should be given as to whether and how this participant is followed in the trial as well as what data has been collected from the participant. The volunteer may be excluded on their own request or as a result of violation of protocol, or if unforeseen circumstances mean that withdrawal from the study is in the participant’s best interest. Unblinding regarding anaesthesia type is permissible if necessary, for safety or medical reasons.

### MRI

All MRI scans will be performed with a 3-Tesla Philips Achieva d-stream with a 32-channel receive head coil. Each MRI scan consists of the following sequences: T1-weighted 3D anatomical MRI (T1w3D), diffusion tensor imaging (DTI), resting-state functional MRI (rsfMRI), BOLD functional MRI measurements after visual stimulation with checkerboard (block design), arterial spin labelling (ASL), Phase Contrast Mapping (PCM), MR susceptometry, MR spectroscopy, and technical scans (e.g. survey scan, B0 field map) needed to obtain sufficient data for performing analysis on the primary scans.

### Cognitive performance tests

The Test of Attentional Performance (TAP) in this study consists of Crossmodal Integration, Go/Nogo, Incompatibility and Flexibility. They measure cognitive integration, attention and flexibility in response to auditive and visual stimuli (TAP 2.3.1 12/2016 from Psytest, D-52134 Herzogenrath). The Paced Auditory Serial Addition Test (PASAT) measures auditive working memory [52]. Conners Continuous Performance Test, 3rd edition (CPT3) measures persistent attention (CPT3, MHS 2014).

### Quality of Recovery-15 Questionnaire and Multidimensional Fatigue Inventory

The volunteers fill in the validated Quality of Recovery Questionnaire (QoR-15) [43] and the Multidimensional Fatigue Inventory (MFI-20) [42]. The QoR-15 questionnaire results in a score of 0–150. The MFI-20 covers aspects of general fatigue, physical fatigue, mental fatigue, motivation and activity in 20 statements with five-scale ratings of agreement.

### Data collection and monitoring

MRI data will be kept on encrypted hard drives behind double-locked doors, as well as on a logged hard drive at the facilities of the Capital Region of Denmark. All other data will be registered in an encrypted, double-locked electronic case report form (eCRF) for each volunteer included in the study. Any AEs or effects will be registered in the eCRF and reported to the sponsor and relevant authorities. All data will be handled in compliance with General Data Protection Regulation (GDPR) and Danish law. The study will be monitored and audited by the Good Clinical Practice (GCP) units in Denmark [53]. Data will be destroyed after 10 years.

### Data processing – MRI

T1-weighted 3D anatomical images will be pre-processed using the FreeSurfer imaging analysis suite (http://surfer.nmr.mgh.harvard.edu/). Other software packages and approaches for data analysis may also be applied. Diffusion-weighted images will be pre-processed using the FSL software package (http://www.fmrib.ox.ac.uk and http://fsl.fmrib.ox.ac.uk/fsl/fsl4.0/tbss/index). This includes inspection of image quality. The resulting data will then be processed via dtifit and tract-based spatial statistic. The re-aligned images will then be fed into bedpostx for local modelling of diffusion parameters. Using bedpostx in conjunction with track-based statistics (TBSS) allows us to model for crossing fibres. The re-aligned images will be fed into probtrackx2 for tractography between the anatomical areas mentioned above. Other software packages and approaches for data analysis may also be applied. Resting-state fMRI (rsfMRI) scans will be analysed using FSL MELODIC (http://fsl.fmrib.ox.ac.uk/fsl/fslwiki/MELODIC). Pre-processing steps will include spatial smoothing, motion correction, slice time correction, high-pass filtering and brain extraction. Individual component analysis (ICA) using data-defined and no less than 60 ICAs will be applied. RsfMRI scans will be co-registered initially to the individual T1w3D anatomical scans (after neutral flipping and brain extraction) and subsequently to MNI 152 brain atlas. A full quality assurance (QA) will be performed consisting of inspection of head movement (< 3 mm and < 3°), acceptable co-registration and inspection of image quality. Resting-state networks (RSNs) will be identified using dual regression. Noise reduction will be performed manually. Randomise command will be performed with a design matrix consisting of the paired *t*-tests with and without co-variates, i.e. age and gender. Results will be displayed upon the MNI-152 normal brain atlas after dimension change as *p* < 0.05 after correction for multiple comparison. Activated areas will be identified semi-automatically. Additional software and methods for data analysis may be applied. In addition, age, gender, physiological variables, blood tests and cognitive test scores, etc. may be used as co-variates in the analysis.

### Statistical analysis

The distribution of continuous data is rated for normality using residual-plot or by the Shapiro-Wilk W test. Normality can be reached by simple transformation (log, logit). Statistical comparison of paired data is performed with parametric or non-parametric method or by analysis of variance (ANOVA). Depending on the distribution, testing is performed with Mann-Whitney or Wilcoxon tests, respectively. The risk for Type I errors is reduced by reducing the significance level to 0.05 (alpha). A general summation goal will be used to avoid mass-significance by repeated measurements. In statistical measurements where multiple comparisons cannot be avoided, Duncan’s corrections (i.e. secondary outcome parameters) are performed. For MRI data, correction for multiple comparisons is performed with Bonferroni’s correction or the false discovery rate (FDR). For statistical comparison between categorical variables (i.e. psychometric data) the *X*^2^ test with Yates’ correction is performed.

### Missing data

For all analyses, an intention-to-test (ITT) analysis will be performed including all subjects that participated in at least one study session. Analyses will be based on all observed data. If missingness exceeds 5% and there is indication of violation of ‘missing completely at random’ (MCAR) by a statistically significant Little’s test, a sensitivity analysis based on an appropriate model for missingness at random (MAR) or not at random (MNAR) will be performed.

### Sample size

We base our sample size estimations on the following primary end points: (1) changes in grey-matter volume, and (2) DTI changes. The volunteers will serve as their own controls.

(i) *Sample size estimation based on changes in grey-matter volume*: we base our sample size estimation for grey-matter volume changes on data extrapolated from the hippocampus. The mean volume of the hippocampus is approximately 2.50 mL (± 0.15 standard deviation (SD)) in the healthy population [54]. The pre-operative hippocampal volume has been found to be 6% smaller in patients suffering from POCD compared to patients not suffering from POCD [55]. We assume that GA will affect the grey-matter volume to a larger extent compared to e.g. vulnerability towards POCD. Thus, 25 subjects will be necessary to detect a difference of 8% in the hippocampal volume between groups, applying a two-tailed alpha value of 0.05, SD ± 10% and a desired power of 0.80
(ii) *Sample size estimation based on DTI changes*: our sample size estimation for detection of DTI changes is also based on data extrapolated from the hippocampus. We assume a 10% change in mean diffusivity, since we expect GA to induce larger changes than, e.g. a learning task by video gaming [56]. With a significance level of 0.05, a power of 80%, a SD of ± 10%, this indicated a required sample size of 16 volunteers

#### Correction for multiple comparisons

Since it should be assumed that grey-matter changes and changes in DTI are inter-dependable variables, the significance level for each sample size estimation should be corrected for multiple comparisons. The desired corrected significance level is 0.025 (significance level of 0.05 Bonferroni corrected for two number of hypothesis). With a SD of ± 10% and 80% power, this gives a sample size of 30 volunteers regarding detection of statistically significant differences in grey-matter volume. Regarding statistically significant differences in DTI, the calculation gives a sample size of 20 volunteers. We are therefore confident that a sample size of 30 volunteers will be sufficient to ensure the necessary statistical power.

### Detection of differences between general anaesthesia methods

For detection of differences between GA methods, we estimate a 10% difference between the two GA methods with regard to both grey-matter changes and changes in DTI. With a significance level of 0.025 (Bonferroni corrected), SD ± 10% and a power of 80%, the sample size is 20. We therefore choose to include 30 volunteers to ensure sufficient power.

### Dissemination of results

The results of this study will be communicated in the form of manuscripts to be submitted for publication in medical journals. Authorship will be according to the Vancouver criteria for authorship as cited in the ICMJE [57]. The results are also expected to be presented at conferences and meetings, as well as made accessible for the public in relevant media. Negative or positive, and conclusive as well as inconclusive results will be published. The public title of this protocol will be ‘*Changes in the brain after GA*’*.*

## Discussion

General anaesthesia is very safe for healthy young adults. In the population as such, mortality related to GA in Denmark is estimated to be 1/100,000–500,000 [58]. The mortality is related to acute procedures especially in the elderly and/or acute or chronically ill patients with serious co-morbidities. The risk will be significantly lower in this study, since the inclusion and exclusion criteria ensure that only healthy young adults without known diseases or risk factors will be included, and a strict focus on safety and quality will be the first priority in carrying out every step of this study. The risk of adverse effects and reactions is very low, and participation in the study is not expected to result in harm or inconvenience. Throughout the planning phase we have focussed on ensuring safety, feasibility and reproducibility. Some relevant choices in the process have been: (1) airway management (laryngeal mask vs. endotracheal tube), (2) anaesthesia induction (and corresponding choice of medication) and (3) IV fluid administration (if needed).

Regarding (1) airway management: the subjects are anaesthetised in a safe environment and a laryngeal mask could have been chosen as the airway device. However, in our opinion, the endotracheal tube offers some advantages over the laryngeal mask. First, we considered it to secure the airway more thoroughly than the laryngeal mask; in case of any unexpected events during GA, the airway will already be secured. Secondly, there will not be any accidental leak of sevoflurane. Finally, an endotracheal tube is a very strong irritant to the lower airway, the acceptance of which ensures that the subjects have reached a sufficient anaesthesia depth. Thus, with careful and skilled intubation and extubation, a GA of sufficient depth, and a close monitoring of our subjects, we find endotracheal intubation to be the best choice in this study.

Regarding (2) induction type: the subjects in this cross-over study are blinded to anaesthesia type. Since the administration of sevoflurane (inhalation of gas) and propofol (IV injection/infusion) are different per se, an induction depending on the anaesthesia type would unblind the subjects, and, thus, reduce the reproducibility between the study sessions. Therefore, we standardised the induction phase regardless of randomisation. We settled on IV induction rather than inhalation induction in these young adults in order to achieve quick and efficient attenuation of airway reflexes, as well as GA of sufficient depth for endotracheal intubation. With propofol as the anaesthetic agent for the induction phase, we could reduce the number of different anaesthetic agents and, thus, avoid introducing additional confounding factors. Since the intubation procedure requires attenuation of airway reflexes, we found it appropriate to administer remifentanil (a fast-acting and efficient opioid). The short half-life of remifentanil compared to the relatively long duration of the GA (2 h) would render any effects observed during or after anaesthesia more likely to be due to sevoflurane and/or propofol rather than to remifentanil. Alternatives to remifentanil would have been very deep propofol sedation (at the risk of profound circulatory depression), a different opioid (which would typically have a longer half-life), or a neuromuscular relaxant (which would also have constituted a confounder, albeit of a different type).

Regarding (3) IV fluid administration: In this study we included healthy subjects only. At our hospital, NaCl 9 mg/ml is the standard vehicle for remifentanil, and for flushing of IV catheters. The amount of fluid to be infused in this study is relatively small (a maximum of 500 ml in total), and although fasting, the subjects are not dehydrated or in need of fluid resuscitation. We thus expect that the relatively small amount of infused sodium and chloride during anaesthesia will be outbalanced during the post-anaesthesia phase.

This protocol represents our best effort to plan and execute a trial that will meet our basic intention with this project. The study should provide crucial information on the mechanisms whereby anaesthetics affect brain function, as well as potential differences between two commonly used types of anaesthetics, sevoflurane and propofol. In addition, hopefully the findings will prove helpful in understanding the physiology and pathophysiology of consciousness.

## Trial status

The protocol H-18028925 (version 2 from 2 July 2019) was approved by the Research Ethics Committee in the Capital Region of Denmark on 17 August 2019. Recruitment began on 6 September 2019. The first participant was enrolled on 9 September 2019. The study is expected to be completed with end of trial for the last participant no later than 31 December, 2020. Any protocol modifications will be reported to the Research Ethics Committee in the Capital Region of Denmark, the GCP unit and, if relevant, the Danish Medicines Agency and EudraCT.

## Supplementary information

**Additional file 1.** SPIRIT 2013 Checklist: Recommended items to address in a clinical trial protocol and related documents.

## Data Availability

The datasets used and/or analysed during the current study are available from the corresponding author on reasonable request, considered compliant with General Data Protection Regulation (GDPR) and the data-sharing agreement is approved by the relevant Danish authorities.

## Abbreviations

AE: Adverse event
ALAT: Alanine transaminase
ASA: American Association of Anesthesiologists
ASL: Arterial spin labelling
BIS: Bispectral index
BMI: Body mass index
BOLD: Blood-Oxygen-Level-Dependent
CMRO_2_: Cerebral metabolic rate of oxygen
CNS: Central nervous system
CPT3: Conner’s Continuous Performance Test, 3rd edition
CRF: Case report form
CRP: C-reactive protein
DASAIM: Danish Society of Anaesthesiology and Intensive Care Medicine
DOB: Date of birth
DTI: Diffusion tensor imaging
ECG: Electrocardiogram
EEG: Electroencephalogram
ETCO_2_: End-tidal CO_2_
fMRI: Functional magnetic resonance imaging
GA: General anaesthesia
GABA: Gamma-aminobutyric acid
GDPR: General Data Protection Regulation
HRV: Heart rate rariability
IL: Interleukin
INR: International normalised ratio
IV: Intravenously administered
LDH: Lactate dehydrogenase
MAC: Minimum alveolar concentration
MFI-20: Multidimensional fatigue inventory
MRI: Magnetic resonance imaging
mRNA: Messenger ribonucleic acid
OEF: Oxygen extraction fraction
PASAT: Paced Auditory Serial Addition Test
PBMC: Peripheral blood mononuclear cells
PCM: Phase Contrast Mapping
PEEP: Positive end-expiratory pressure
POCD: Post-operative cognitive dysfunction
POD: Post-operative delirium
RF: Respiratory frequency
RNA: Ribonucleic acid
rsfMRI: Resting-state functional magnetic resonance imaging
SAE: Serious adverse event
SARI: Simplified airway risk index
SpO_2_: Oxygen saturation
TAP: Test of Attentional Performance
T1w3D: T1-weighted three-dimensional
TBSS: Tract-based statistics
TCI: Target-controlled infusion
TIVA: Total intravenous anaesthesia
TNF: Tumour necrosis factor
VBM: Voxel-based morphometry
